# Dataset of conditions and characteristics of street vendors located in public spaces in Colombia

**DOI:** 10.1016/j.dib.2023.109689

**Published:** 2023-10-15

**Authors:** Johanna Peláez-Higuera, Gregorio Calderón-Hernández, Héctor-Mauricio Serna-Gómez

**Affiliations:** Universidad de Manizales, Manizales, Colombia

**Keywords:** Informal economy, Street trade, Intermediate city, Florencia – Caquetá, Dataset, Cartography

## Abstract

The objective of this document is to introduce the datasets and the methods for accessing them, derived from the article “Social, commercial, and economic diversity. Poverty and expectations among street vendors in Florencia, Caquetá, Colombia.” These datasets aim to provide insights into the conditions and characteristics of street vending in Colombia. The data collection process involved both mapping and personal surveys conducted on 190 street vendors. Additionally, practical recommendations are provided for tailoring the implementation of each survey instrument based on the specific attributes of the study's target demographic. The collected data holds the potential for comparative and longitudinal analyses, not only within different Colombian cities but also in cities worldwide facing similar circumstances to those of intermediate cities like Florencia. These datasets offer a valuable resource for understanding the dynamics of street vending and its implications, fostering more comprehensive research and informed policymaking.

**Table:** Data Specification

Subject	Informal Economy
Specific subject area	Street vending. Street vending is a form of informal economy. In low- and middle-income countries, it is often a source of income for vulnerable communities, while in high-income countries, it is an employment mechanism for the immigrant population.
Data format	Raw
Type of data	Table
Data collection	The information was gathered from 190 street vendors located in public spaces in Florencia, Caquetá, Colombia, which corresponds to 60.13% of the 316 vendors located in the city's downtown area. We use cartography, and questionnaires. The only condition for participating in the survey was the street vendor's decision. For the questionary we used 16 questions. For cartography the street vendor public space localization.
Data source location	The data was collected in Florencia, Caquetá, Colombia, South America. The data is stored In Mendeley [1].
Data accessibility	Repository name: Mendeley Data identification number: 10.17632/ygbypnb8zg.5 Direct URL to data: https://data.mendeley.com/datasets/ygbypnb8zg/5
Related research article	[2] Peláez-Higuera, J. Calderón-Hernández, G. Gómez-Serna, H-M. Social, commercial, and economic diversity. Poverty and expectations among street vendors in Florencia, Caquetá, Colombia. Cities, 140, (2023) 1-10-. https://doi.org/10.1016/j.cities.2023.104448

## 1 Value of the Data

• The data were collected in an intermediate city with special conditions of forced displacement and push-and-pull characteristics.
• The data correspond to a developing line of work through which it is hoped knowledge related to other intermediate cities will continue to be generated, with these data and the related article being the foundation for providing follow-up to the study on street vending in Colombia.
• The data can contribute to the creators and executors of public policies to generate pertinent solutions.
• The data could be used for comparative analysis between countries and cities with conditions like those of Florencia Caquetá Colombia.
• This data could be used to analyze and understand the main differences between sectors of the informal economy.
• Informal vendors are a difficult population to survey, since answering implies neglecting commercial activity, so this information could be used to understand the phenomenon of street vending.
• The data establishes the interest of street vendors in entering the formal labor market, including the salary they would be willing to receive.

## 2 Data Description

In the data provided in the excel file available in Mendeley [1], there are 21 columns with data that state the following conditions:

**Table 1:** Data Codification.

Codification	%
***Gender***	

Man	46.3%
Woman	53.7%

***Age group***	

Less than 18	3.7%
Between 18 y 30	23.7%
Between 31 y 60	67.6%
Over 60	10.5%

***Marital status***	

Single	27.4%
Married or Free Union	50.5%
Separated, divorced, widowed	22.1%

***Sisbén***	

1	64.7%
2	3.2%
3	1.6%
No information, don't know	30.5%

***Socioeconomic Level***	

1	76.3%
2	8.4%
3	4.2%
No information, don't know	11.1%

***Product marketed***	

Food and beverages	57.9%
Entertainment and culture	5.2%
Jewellery and perfumes	15%
Home	12.1%
Clothing	9.8%

***Place of Origin***	

Florencia	22,1%
Other places	64.7%
No information	13.2%

***Reason to change housing location***	

threat or risk to your life	42,2%
family reasons	4,7%
Work	42,2%
Other reason	10,9%

***Can read and write***	

Yes	92,1%
No	6,8%
no response	1,1%

***Educational level***	

None	8.4%
Some year of primary school	40%
Some year of high school	36.9%
Some year of higher education	8.4%
no response, no information, don't know	6.3%
***Perception of low household income***	

Yes	61.1%
No	36.3%
No response	2.6%

***Are you satisfied with your location in the public space?***	

Yes	78,4%
No	20,5%
No response	1,1%

***How would you be affected by a relocation?***	

Benefited	13,7%
Injured	38,9%
Neither one nor the other	24,7%
Do not know do not answer	22,6%

***Have you thought about changing your job?***	

Yes	56,8%
No	43,2%

***Do you think you can find another job?***	

Yes	43,7%
No	56,3%

***If you were offered a job with your current income and social security, would you accept?***	

Yes	77,4%
No	21,6%
no response	1,1%

***Salary you would accept to stop street vending***	

Less than 1 legal monthly minimum wage	15,8%
Between 1 and 2 legal monthly minimum wage	73,2%
More than 2 legal monthly minimum wage	3,7%
Would not accept	4,2%
Don´t know	3,2%

## 3 Experimental Design, Materials and Methods

The data collection process took place in Florencia, Caquetá, Colombia during the months of September and October 2018.

- Population

The population corresponded to street vendors located in public spaces, who were selling their products at the time of the data collection. No vendor was discriminated against because of age or product; the only condition for participating in the research was their willingness to provide data.

- Instruments and data collection

The first data collection instrument was mapping. For its use, the area with the greatest presence of street vendors—corresponding to 10^–^14th Avenues and 13^–^16th Streets—was selected. Starting there, a georeferencing map was created and subsequently used to go through all the avenues and streets, where five groups of products were identified: food and drink, entertainment and culture, jewelry and perfumes, household, and clothing. A different color was used for each of these products, and the vendor was positioned according to the address on the map. This mapping was verified at the end of data collection for greater accuracy, and it was used to identify 316 vendors located solely in the downtown area [2]. It is important to mention that the mapping served as a tool for the selection of the minimum number of vendors to be surveyed to increase the reliability and representativeness of the data. This mapping also made it possible to analyze the areas with the greatest influx of street vendors and the types of products most sold.

**Fig. 1:**
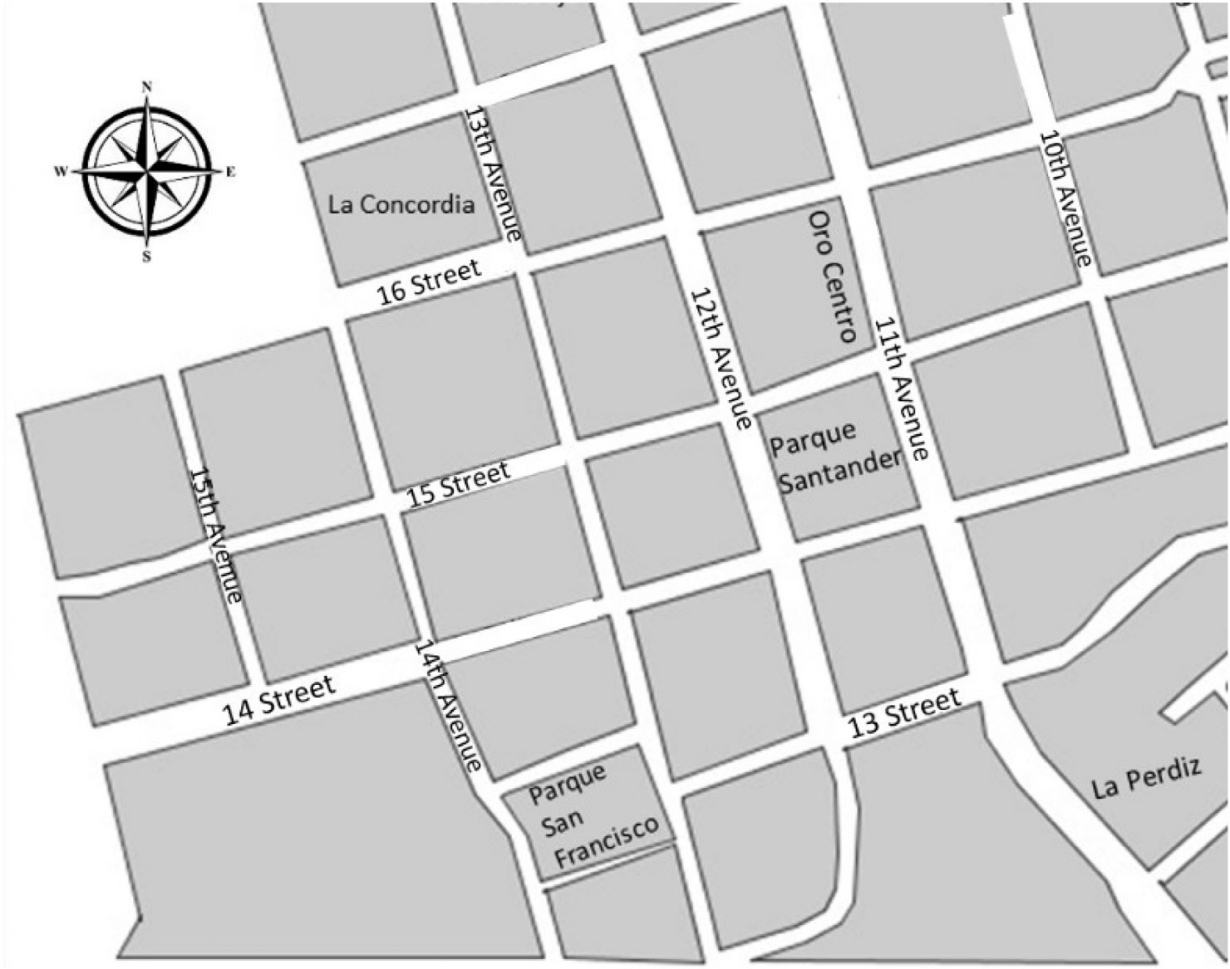
Mapping. **Source**: Prepared by the authors.

During the mapping, the 316 vendors were classified by five product types, finding that, as in the survey results, food and beverage products represent the most widely distributed product type; in second place, household products; in third place, clothing and footwear; in fourth place, jewellery and perfumes; and finally, in fifth place, entertainment and culture. this information helps to establish possible biases in the information between the survey and the mapping.

**Table 2:** Description of mapping results.

CENSUS		
***Product marketed***	***#***	***%***
Food and beverages	169	53,5%
Entertainment and culture	27	8,5%
Jewellery and perfumes	36	11,4%
Home	45	14,2%
Clothing	39	12,3%

The second instrument used was a survey, containing six modules and which had been used previously. For the purposes of the article “Social, commercial, and economic diversity. Poverty and expectations among street vendors in Florencia, Caquetá, Colombia,” 21 questions were used. For the collection of these data the same person who prepared the mapping undertook to visit each of the street vendor stalls to invite possible participation in the study.

The collection of information was carried out under a systematic framework. Although it is true that informal vendors are stationary - with a permanent location for their work -, semi-stationary - with a temporary location for their work, e.g. candy carts -, and mobile - with a permanent movement of their work setup -, all of them are confined to a specific work zone, which allows for the systematic recognition of their location.

Considering this, a preliminary survey of the city's streets and roads with the highest concentration of informal sales was conducted. Subsequently, using a systematic approach with fixed survey schedules, individual vendors were invited for participation, with each vendor located on specific streets. The systematic structure involved moving through each of the identified streets for the study, and after completing the survey process for one street, moving on to the next.

Following this, a further round was conducted in the previously surveyed streets to identify potential vendors who hadn't been initially invited to participate in the study. The aim was to achieve the highest possible participation rate in the study.

The systematic nature of this procedure allowed for the collection of information from 60,1% of the total population. The proportion of the population not surveyed is due to vendors declining to participate in the study, primarily driven by institutional distrust.

For application of the instrument, a survey manual was also created presenting the guidelines for approaching the street vendors and the possible responses that could be obtained, as well as the length of each question, how to treat the street vendors and the respect that must be present in each interaction, keeping in mind the vendors’ characteristics and cultural aspects of this population.

The quantitative data collection processes required an understanding of the vendors’ conditions, to the extent that they were at their workplace and interaction with the surveyor had to stop when a client was present. For this reason, when the team of surveyors were introducing themselves and mentioning the conditions and intentions of the survey, the clarification was offered, that as soon as the survey had to be stopped, this would be respected so as not to interfere with their principal activity.

Likewise, the vendors’ attitudes were quite generous overall, as those who sold perishables offered their product for free, and rejecting their offer would seem rude to them, since they were also attempting to make the information collectors feel at ease. A few vendors gave the condition of buying a product from them or giving them some kind of monetary incentive in exchange for the information; this was rarely accepted, since it jeopardized the trustworthiness of the data.

**Fig. 2:**
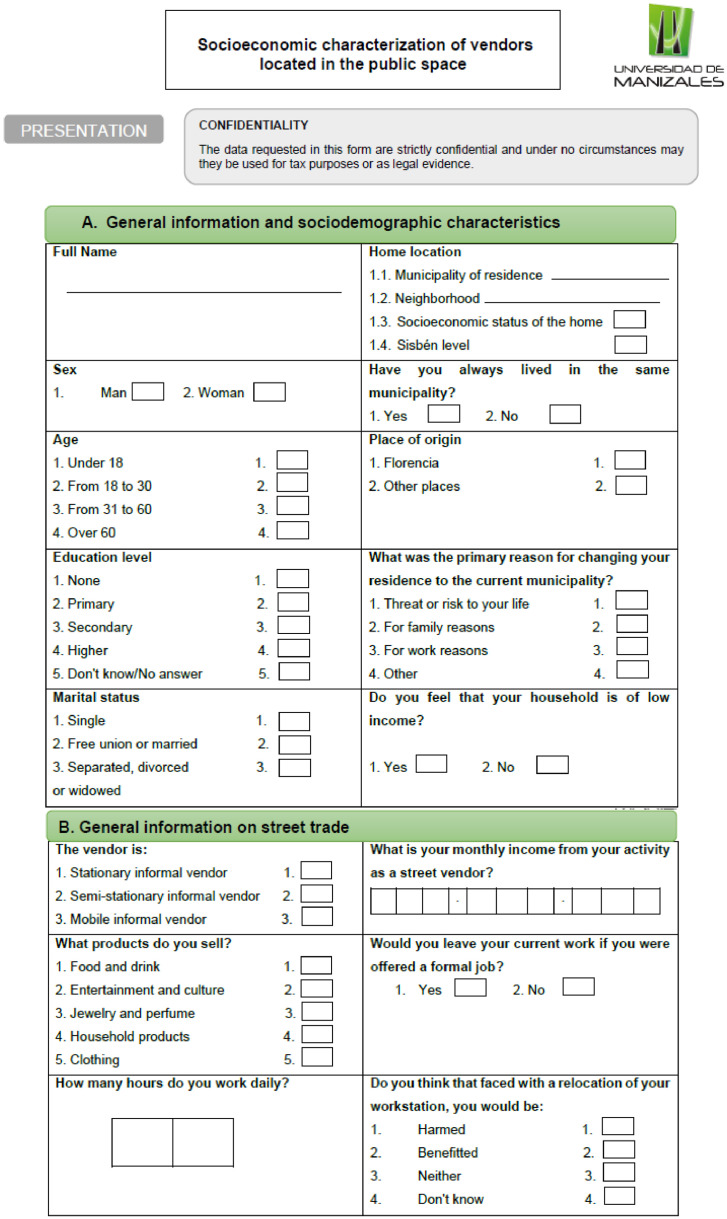
Survey for quantitative data collection.

The questions used for this questionnaire are part of a larger instrument. For the purposes of this research, the most relevant questions were used to understand the phenomenon of street vending in the territory.

- Tools for data processing

For data processing, the data was first tabulated in Excel. SPSS was used to perform clustering, descriptive analysis, significance analysis and standard deviations, based on the variables available in the excel file.

## Limitations

Not applicable.

## Ethics Statement

For the dataset all the subjects were asked for their informed consent. We declare that we did not conduct any experiments and all data have been full anonymized.

For this research, no endorsement from the bioethics committee was requested since the Universidad de Manizales does not request this condition because we did not collect sensitive data, and the information was treated with anonymity.

It is important to mention that in the case of the two minors who participated in the study, they were delegated directly by their parents because they considered that due to their level of education, the young person would have a greater capacity to respond, additionally, the parents were with them at the time of the survey.

We also state that the data is confidential in that the respondent's name is not identified in the database, only the respondent is listed, and additionally, respondents were informed that this information could only be used for academic purposes. The information obtained from street vendors is not for tax or legal purposes.

## Declaration of Competing Interest

The authors declare that they have no known competing financial interests or personal relationships that could have appeared to influence the work reported in this paper.

## Declaration of Generative AI and AI-assisted Technologies in the Writing Process

During the preparation of this work the author(s) used CHAT GPT to improve the translation of the original ideas. After using this tool/service, the author(s) reviewed and edited the content as needed and take(s) full responsibility for the content of the publication.

## CRediT authorship contribution statement

**Johanna Peláez-Higuera:** Conceptualization, Writing – original draft, Methodology, Writing – review & editing. **Gregorio Calderón-Hernández:** Conceptualization, Writing – original draft, Methodology, Writing – review & editing. **Héctor-Mauricio Serna-Gómez:** Conceptualization, Methodology, Writing – review & editing.

## Data Availability

Street vendors in Florencia-Caqueta-Colombia (Original data) (Mendeley Data)
